# A Case of Poisoning by Nicotina

**Published:** 1869-08

**Authors:** 


### A case of Poisoning by Nicotina.

The following case of poisoning by nicotina may perhaps, on account of the manner in which it was produced, prove interesting and instructive. I was summoned on the 21st instant to see a little boy, seven years of age, said to be in a fit. On arriving at the house I found him completely insensible, cold, pulseless, with prolonged respiration. On trying to rouse the child, I discovered a blackish patch, about the size of the palm of the hand, on the side of his neck, which I was informed was ringworm, and that an ever-ready old woman prescriber, with which this neighborhood is blessed, had advised the parents to procure an old much-used tobacco pipe, to scrape its interior, and apply the ash, mixed with a little oil, to the abraded surface. In the course of half an hour the child went to his father complaining of a sense of choking, tottering in his gait, and vomiting. I saw him about twenty minutes after, and found him in the state above described. The father assured me the quantity of ash applied could be held on the point of a tolerable-sized penknife. The treatment pursued was, having the part immediately well washed with soap and water, rousing the little patient, administering ammonia and coffee, with friction to the limbs, &c. Consciousness and reaction soon commenced returning, and in an hour or so the child was out of danger.—*Lancet.*

